# Obestatin Accelerates the Recovery in the Course of Ischemia/Reperfusion-Induced Acute Pancreatitis in Rats

**DOI:** 10.1371/journal.pone.0134380

**Published:** 2015-07-30

**Authors:** Jakub Bukowczan, Zygmunt Warzecha, Piotr Ceranowicz, Beata Kuśnierz-Cabala, Romana Tomaszewska

**Affiliations:** 1 Department of Endocrinology and Diabetes Mellitus, Northumbria NHS Foundation Trust, Rake Lane, North Shields, Tyne and Wear, United Kingdom; 2 Department of Physiology, Jagiellonian University Medical College, Krakow, Poland; 3 Department of Clinical Biochemistry, Jagiellonian University Medical College, Krakow, Poland; 4 Department of Pathology, Jagiellonian University Medical College, Krakow, Poland; University of Szeged, HUNGARY

## Abstract

**Objective:**

Several previous studies have shown that obestatin exhibits protective and regenerative effects in some organs including the stomach, kidney, and the brain. In the pancreas, pretreatment with obestatin inhibits the development of cerulein-induced acute pancreatitis, and promotes survival of pancreatic beta cells and human islets. However, no studies investigated the effect of obestatin administration following the onset of experimental acute pancreatitis.

**Aim:**

The aim of this study was to evaluate the impact of obestatin therapy in the course of ischemia/reperfusion-induced pancreatitis. Moreover, we tested the influence of ischemia/reperfusion-induced acute pancreatitis and administration of obestatin on daily food intake and pancreatic exocrine secretion.

**Methods:**

Acute pancreatitis was induced by pancreatic ischemia followed by reperfusion of the pancreas. Obestatin (8nmol/kg/dose) was administered intraperitoneally twice a day, starting 24 hours after the beginning of reperfusion. The effect of obestatin in the course of necrotizing pancreatitis was assessed between 2 and 14 days, and included histological, functional, and biochemical analyses. Secretory studies were performed on the third day after sham-operation or induction of acute pancreatitis in conscious rats equipped with chronic pancreatic fistula.

**Results:**

Treatment with obestatin ameliorated morphological signs of pancreatic damage including edema, vacuolization of acinar cells, hemorrhages, acinar necrosis, and leukocyte infiltration of the gland, and led to earlier pancreatic regeneration. Structural changes were accompanied by biochemical and functional improvements manifested by accelerated normalization of interleukin-1β level and activity of myeloperoxidase and lipase, attenuation of the decrease in pancreatic DNA synthesis, and by an improvement of pancreatic blood flow. Induction of acute pancreatitis by pancreatic ischemia followed by reperfusion significantly decreased daily food intake and pancreatic exocrine secretion. Administration of obestatin at doses used was without significant effect with regard to daily food intake or pancreatic exocrine secretion in sham-operated rats, as well as in rats with acute pancreatitis. On the other hand, obestatin abolished a statistical significance of difference in food intake between animals with AP and control animals without pancreatic fistula and induction of AP.

**Conclusion:**

Treatment with the exogenous obestatin reduces severity of ischemia/reperfusion-induced acute pancreatitis and accelerates recovery in this disease. The involved mechanisms are likely to be multifactorial, and are mediated, at least in part, by anti-inflammatory properties of obestatin.

## Introduction

Acute pancreatitis (AP) is the most common pancreatic disease in clinical practice [1–3]. Despite substantial improvements in the management of the disease over the last decade, AP still remains associated with high morbidity and mortality rates reaching up to 30% in severe cases [4,5]. This is mainly due to its complex etiology and clinical course, as well as, the lack of targeted treatment for pancreatitis owning to the poor understanding of its pathogenesis. A number of pathophysiological processes including inflammation, apoptosis, necrosis and oxidative stress have been associated with AP and are responsible for irreversible morphological and structural changes of the gland in the course of severe AP [6].

Obestatin is a circulating 23-amino-acid peptide, encoded by the same gene as ghrelin [7]. It is predominantly produced in the stomach, and exhibits a wide range of peripheral effects including inhibition of food intake, body weight gain, gastric emptying and regulation of jejunal motility [7–9]. Obestatin expression has been also found in in the endocrine pancreas, where it is colocalized with ghrelin in fetal and adult human pancreas. Moreover, obestatin is secreted by pancreatic β-cell lines and human pancreatic islets [8, 9]. Incubation of pancreatic β-cell line, INS-1E, as well as human islets with anti-obestatin antibody has been shown to reduce cell viability, suggesting that obestatin may act in the pancreas through autocrine/paracrine mechanisms [7–9]. Interestingly, several previous studies have demonstrated the protective and regenerative effects of the preproghrelin gene-derived peptides, including obestatin and ghrelin in the gastrointestinal tract, kidney and brain [10–12]. Likewise, a positive effect of obestatin has also been observed in the pancreas, where the peptide exhibited protective action in cerulein-induced AP [13]. In addition, it has been shown that obestatin promotes survival and proliferation, and prevents apoptosis in both β-cells and human islets of the pancreas [8,14,15]. Furthermore, obestatin-induced modulation of FGFR/Notch/Ngn3 developmental pathways, together with its expression in fetal pancreas, indicates its involvement in the gland formation and organ regeneration [8,16].

Although, protective properties of obestatin against the cerulein-induced AP have been proved, it still remains unknown whether this peptide exerts therapeutic effect in the course of AP. Therefore, the aim of this study was to investigate whether administration of obestatin affects the course of pancreatitis in a rat model of ischemia/reperfusion-induced AP.

## Material and Methods

### Animals and treatment

All studies followed an experimental protocol and both the Institutional Animal Care and Use Committee (IACUC) at the Jagiellonian University, as well as the Local Ethics Committee specifically approved this study.

The experiments were carried out on adult male Wistar rats, weighing 200-270g (the first series of studies) or 280-320g (the second series of studies). During the experiment, animals were housed separately in wire-mesh bottom cages, temperature was adjusted at 22±1°C with relative humidity of 50 ± 10%, and 12h:12h light:dark photoperiod. After one-week period of acclimation to their new environment, experiments were carried out in two separate series. The first series of studies were performed to determine the influence of obestatin administration on the recovery in the course of ischemia/reperfusion-induced AP. The second series of studies were performed to check the influence of administration of obestatin on food intake, as well as to determine the effect of the development of ischemia/reperfusion-induced AP and treatment with obestatin on pancreatic exocrine secretion. At the each endpoint of studies, animals were anesthetized with ketamine (50mg/kg intraperitoneally, Bioketan, Vetoquinol Biowet, Gorzów Wielkopolski, Poland) and euthanized by exsanguination from abdominal aorta.

In the first series of studies, animals were randomly assigned to 3 experimental groups, as follows: (1) sham-operated saline-treated rats (control group); (2) rats treated with saline after the development of ischemia/reperfusion-induced AP; (3) rats treated with obestatin after the development of ischemia/reperfusion-induced AP. Animals were fasted with free access to water for 24h before induction of AP, food and tap water were available ad libitum later on. In this series of studies, each experimental group in each time of observation primary consisted of 10 rats. During experiment three animals died between the first and second day after induction of AP due to abdominal hemorrhage and ascites leading to circulatory insufficiency. For this reason, three additional animals were used to reach 10 observations in each experimental group and each time of observation.

Surgical procedure for induction of AP by pancreatic ischemia followed by reperfusion was performed according to the method previously described [17]. Briefly, general anesthesia was achieved intraperitoneally with ketamine (50mg/kg, Bioketan, Vetoquinol Biowet, Gorzów Wielkopolski, Poland). Using sterile technique a longitudinal laparotomy was performed, and ischemia in the splenic region of the pancreas was induced by clamping the inferior splenic artery for 30min applying microvascular clips, which were then removed to allow reperfusion, and the abdominal cavity was closed. In sham-operated control animals longitudinal laparotomy and mobilization of the pancreas without artery clamping was carried out.

During the first 2 days of postoperative period, all rats were monitored every 8 h and received subcutaneously tramadol (1mg/kg/dose, Poltram 100, Polpharma, Starogard Gdański, Poland) as an analgetic to minimize pain and distress. Also, at the time 0, 12 and 24 h after surgery, all rats were injected subcutaneously with 10 ml of Ringer’s solution to supplement the loss of fluids during surgery and postoperative period.

Treatment with saline or obestatin was started 24 hours after the beginning of pancreatic reperfusion. Obestatin was administered intraperitoneally twice a day at a dose of 8nmol/kg/dose. This dose of obestatin was chosen because previous studies have shown that obestatin given at the dose of 8nmol/kg/dose exhibits a strong and stable protective effect in the pancreas [13]. Rat obestatin was obtained from Yanaihara Institute (Shizuoka, Japan). The severity of ischemia/reperfusion-induced pancreatitis was assessed on days 1, 2, 5, 9, and 14, respectively, following surgery.

### Determination of pancreatic blood flow

At the each endpoint of studies, the animals were again anesthetized with ketamine, and the abdominal cavity was opened. Pancreatic blood flow in the splenic region of the pancreas was measured by a laser Doppler flowmeter using PeriFlux 4001 Master monitor (Perimed AB, Järfälola, Sweden), as described previously [17]. The data was presented as percent change from value obtained in sham-operated saline-treated rats without induction of acute pancreatitis.

### Biochemical analyses

Immediately following measurement of pancreatic blood flow, blood samples were collected from the aorta, and serum was stored and frozen at -60°C. Serum lipase activity was determined with Kodak Ectachem DT II System analyzer (Eastman Kodak Company, Rochester, NY, USA) using a commercially available Lipa DT Slides (Vitros DT Chemistry System, Johnson & Johnson Clinical Diagnostics, Inc., Rochester, NY, USA). Serum concentration of interleukin-1β (IL-1β) was measured using the commercial BioSource Cytoscreen rat IL-1β kit (BioSource International, Camarillo, California, USA) based on ELISA.

### Myeloperoxidase activity

Pancreatic neutrophil infiltration was determined by histological examination and quantifying tissue myeloperoxidase activity (MPO) [18]. Tissue extraction for evaluation of pancreatic MPO activity, DNA synthesis, as well as the morphological studies followed the protocol described in detail elsewhere [19]. Briefly, during aortic blood samples collection, anesthetized animals were euthanized by exsanguination. The pancreas was carefully dissected out from its attachments to the stomach, duodenum, and spleen. Fat and peripancreatic tissues were trimmed away. Samples of pancreatic tissue from the splenic region of the pancreas were taken for determination of the pancreatic MPO activity, DNA synthesis, as well as the morphological studies. Pancreatic tissue used for determination of MPO activity was homogenized in 0.1 M sodium phosphate buffer containing 0.5% hexadecyl trimethyl ammonium bromide (Sigma-Aldrich, St. Louis, USA) and 5% soybean trypsin inhibitor (Sigma-Aldrich, St. Louis, USA), and then directly frozen on dry ice. The specimens were freeze-thawed three times, and sonicated after each cycle. Suspensions were then centrifuged at 20,000 *g* for 15 min. MPO activity in supernatant was measured with a spectrophotometer at 470 nm by mixing an aliquot (25 μl) of the supernatant with 1.0 ml of 0.1 M sodium phosphate buffer (pH 7.0) containing 0.0016 ml of guaiacol (Sigma-Aldrich, St. Louis, USA) and 0.0005% hydrogen peroxide (Sigma-Aldrich, St. Louis, USA) as substrates. MPO activity was expressed as the percentage of MPO activity obtained in control animals.

### Determination of pancreatic DNA synthesis

The rate of DNA synthesis was measured by incubation of minced pancreatic tissue at 37°C for 45 min in 2ml of medium containing 8μCi/ml of [3H]thymidine ([6-3H]-thymidine, 20–30 Ci/mmol, Institute for Research, Production and Application of Radioisotopes, Prague, Czech Republic), as described previously [20]. DNA concentration in samples was determined by the Giles and Myers method [21]. DNA synthesis was expressed as [3H]thymidine disintegration per minute per microgram DNA (dpm/μg DNA).

### Histological evaluation

Microscopic examination of pancreatic tissue damage was conducted in hematoxylin and eosin stained slides and followed the previously described protocol [22]. Two experienced pathologists, who were blinded to the sample groups, performed histological evaluation (4 slides per animal), and graded edema, leukocyte inflammatory infiltration, vacuolization of the acinar cells, hemorrhages, and necrosis applying a scale ranging from 0 (no changes) to 3 (maximal alterations), as described previously [22]. The results of histological examination have been expressed as a predominant histological score (mode) in each experimental group of animals.

### Assessment of food intake and secretory function

In the first part of the second series of studies, we have the influence of obestatin on daily food intake in intact rats without any operation. Twenty two animals were randomly divided into two equal experimental groups: (1) control rats treated twice i.p. with saline during one-day observation; (2) rats treated twice i.p. with obestatin during one-day observation. Obestatin was given at the dose of 8nmol/kg/dose as in the first series of studies. Amount of food available to animals was checked 1 h before treatment with saline or obestatin, and 24 h later. Difference between both values has been recognized as a daily food intake.

Two days later, animals from the second series of studies were fasted for 24 h and under general anesthesia with ketamine equipped with chronic pancreatic fistula as described previously [23]. After 5 days recovery, rats were divided into four experimental groups: (1) sham-operated saline-treated rats (control group); (2) sham-operated obestatin-treated rats; (3) rats treated with saline after the development of ischemia/reperfusion-induced AP; (4) rats treated with obestatin after the development of ischemia/reperfusion-induced AP.

The first and second group of animals consisted of 5 rat per group. The third and fourth group primary consisted of 6 rats per group. However, during the first day after induction of AP one rat died due to abdominal hemorrhage and ascites leading to circulatory insufficiency. For this reason, finally the third group of animals consisted of 6 rats; whereas the fourth group consisted of 5 rats. Sham-operation and acute pancreatitis were performed likewise in the first series of studies. During of postoperative period after preparation of chronic pancreatic fistula, sham-operation and induction of AP, all rats received subcutaneously tramadol and Ringer’s solution as in the first series of studies.

Secretory studies were performed in conscious rats at third day after sham operation or induction of AP. Before examination of pancreatic exocrine secretion, animals were fasted for 12 h. Animals were treated with saline or obestatin as at the first series of studies. One h after the last i.p. administration of saline or obestatin, rats were placed in individual Bollmann-type cages and pancreatic juice from pancreatic fistula was collected for 30 min to determine the volume and amylase output in basal condition. After that pancreatic secretion was stimulated by cerulein (Sigma-Aldrich GmbH, Steinheim, Germany) given i.p. at the dose of 1 μg/kg and pancreatic juice was collected for next 30 min, starting 30 min after administration of cerulein. Activity of amylase in pancreatic juice was determined as in the first series of studies.

On the fourth day after sham operation or induction of AP, we determined the influence of induction of AP and treatment with obestatin on daily food intake as described above. Then rats were euthanized by exsanguination as animals from the first series of studies.

Statistical analysis of data obtained in both series of studies was performed by one-way analysis of variance (ANOVA) followed by Tukey’s multiple comparison test using GraphPadPrism (GraphPad Software, San Diego, CA, USA). The results were presented as mean values ± standard error of the mean (SEM), and a p value <0.05 was considered statistically significant.

## Results

### Pancreatic blood flow

**Fig 1:**
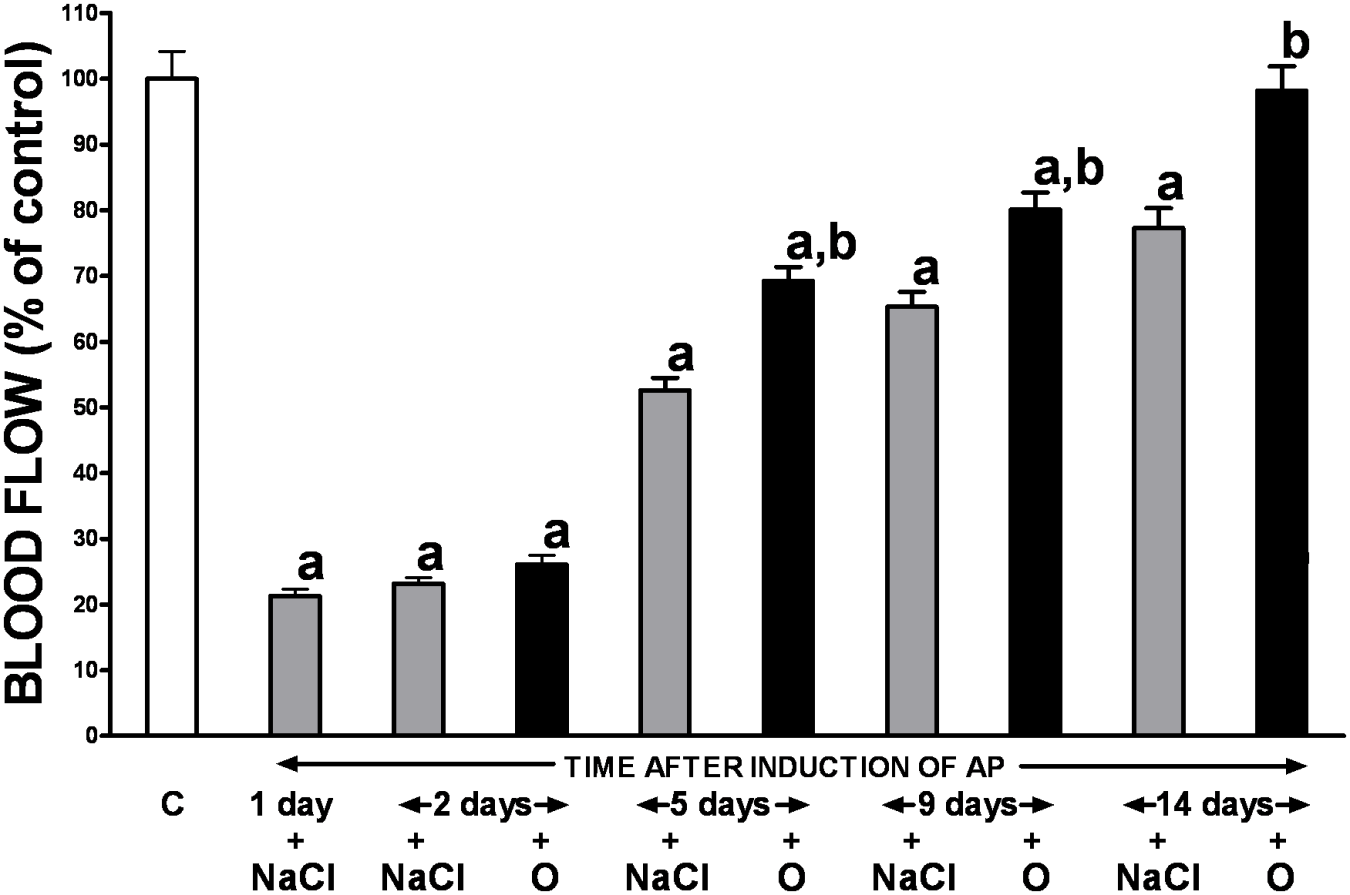
Influence of treatment with saline or obestatin on pancreatic blood flow in the course of ischemia/reperfusion-induced acute pancreatitis. Key: C = control without induction of acute pancreatitis and treated with saline; O = obestatin given i.p. at the dose of 8 nmol/kg/dose twice a day, starting 24 h after induction of acute pancreatitis; NaCl = saline given i.p. twice a day, starting 24 h after induction of acute pancreatitis; AP = ischemia/reperfusion-induced acute pancreatitis. Values are expressed as mean ± SEM. ^a^P<0.05 compared to control; ^b^P<0.05 compared to NaCl-treated rats after induction of AP at the same time of observation.

Acute ischemia/reperfusion-induced pancreatitis resulted in an immediate dramatic reduction of the gland blood flow (by approximately 80%), as compared to the sham-operated saline-treated control group (Fig 1). However, this detrimental effect was not permanent and pancreatic blood flow spontaneously and gradually improved reaching nearly 80% of baseline value on day 14. Obestatin treatment resulted in statistically significant expedition of pancreatic tissue blood flow recovery observed since the 5th day of pancreatic reperfusion, with almost full recuperation achieved 7 days later (Fig 1).

### Biochemical findings

**Fig 2:**
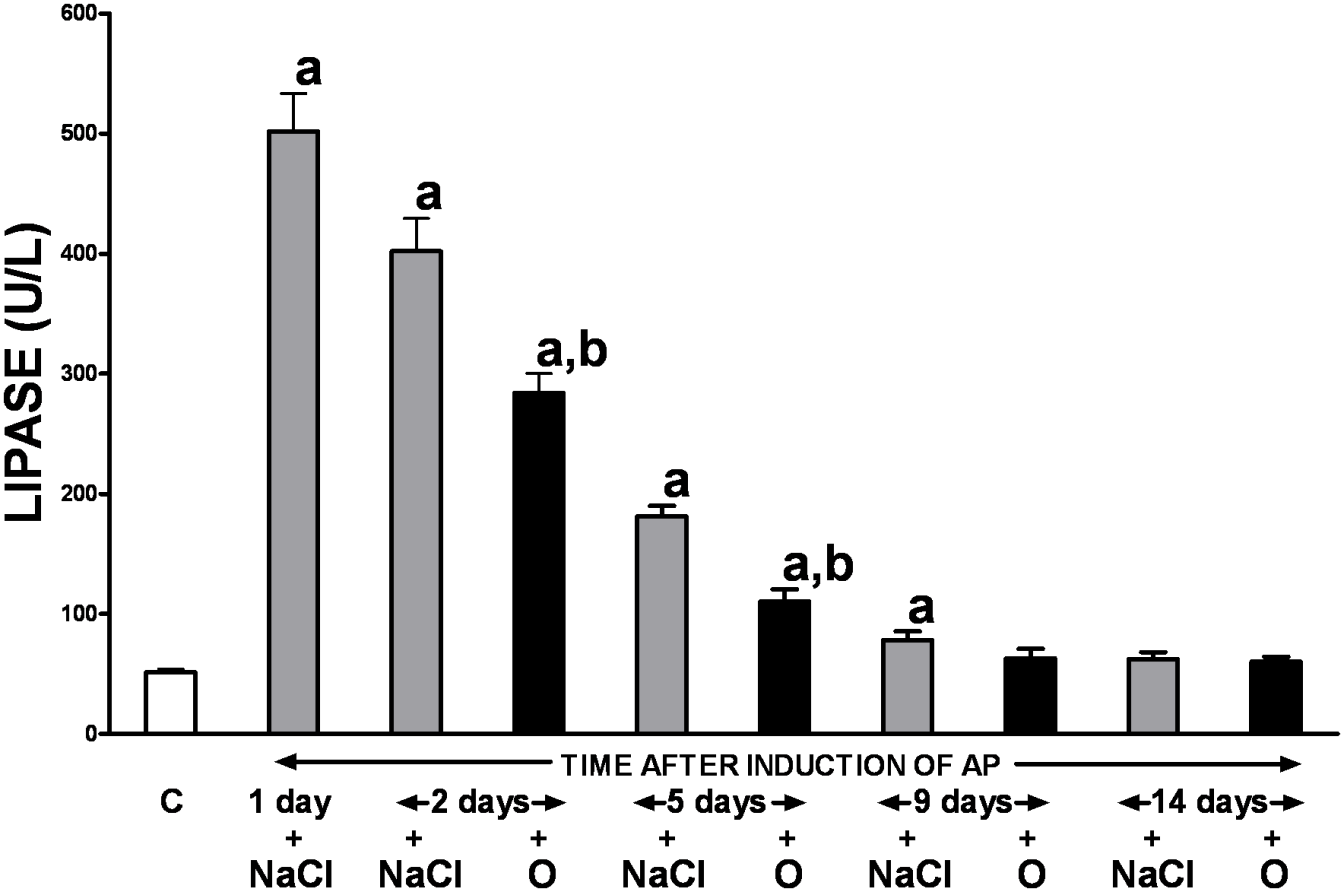
Influence of treatment with saline or obestatin on serum activity of lipase in the course of ischemia/reperfusion-induced acute pancreatitis. Key: C = control without induction of acute pancreatitis and treated with saline; O = obestatin given i.p. at the dose of 8 nmol/kg/dose twice a day, starting 24 h after induction of acute pancreatitis; NaCl = saline given i.p. twice a day, starting 24 h after induction of acute pancreatitis; AP = ischemia/reperfusion-induced acute pancreatitis. Values are expressed as mean ± SEM. ^a^P<0.05 compared to control; ^b^P<0.05 compared to NaCl-treated rats after induction of AP at the same time of observation.

As demonstrated in Fig 2, rats with ischemia/reperfusion-induced pancreatitis produced a 10-fold increase in serum lipase activity compared to the controls. The observed deleterious effect was temporary and the gradual spontaneous decrease in serum lipase activity was observed throughout the study, starting on day 2 of the experiment. The enzyme level close to that in control group was achieved two weeks following surgery. Treatment with obestatin accelerated normalization of serum lipase activity. Statistically significant the obestatin-evoked reduction in enzyme activity compared to the values obtained from animals that received no polypeptide treatment was observed between the 2^nd^ and 5^th^ day of the study, and the baseline lipase activity was achieved on the day 9.

**Fig 3:**
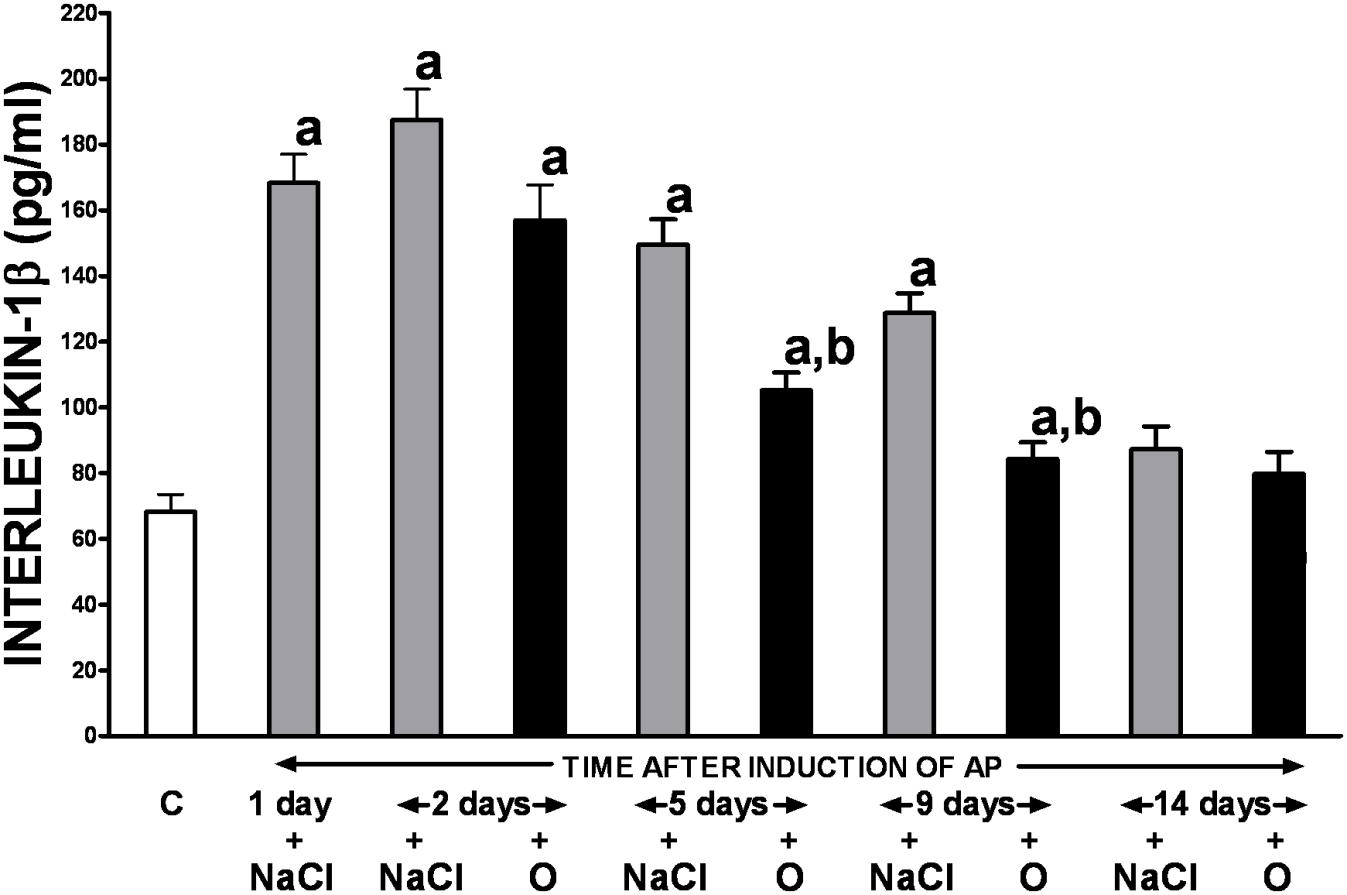
Influence of treatment with saline or obestatin on serum concentration of interleukin-1β in the course of ischemia/reperfusion-induced acute pancreatitis. Key: C = control without induction of acute pancreatitis and treated with saline; O = obestatin given i.p. at the dose of 8 nmol/kg/dose twice a day, starting 24 h after induction of acute pancreatitis; NaCl = saline given i.p. twice a day, starting 24 h after induction of acute pancreatitis; AP = ischemia/reperfusion-induced acute pancreatitis. Values are expressed as mean ± SEM. ^a^P<0.05 compared to control; ^b^P<0.05 compared to NaCl-treated rats after induction of AP at the same time of observation.

Changes in serum concentration of pro-inflammatory interleukin-1β (IL-1β) were similar to the pattern seen in relation to lipase activity (Fig 3). Following the initial increase in IL-1β level immediately after induction of AP, with a maximal nearly 3-fold rise observed on the day 2, there was a gradual normalization of its concentration seen throughout the study. Treatment with obestatin precipitated that process and this effect was statistically significant between the 5^th^ and 9^th^ day of the experiment.

### Pancreatic myeloperoxidase activity

**Fig 4:**
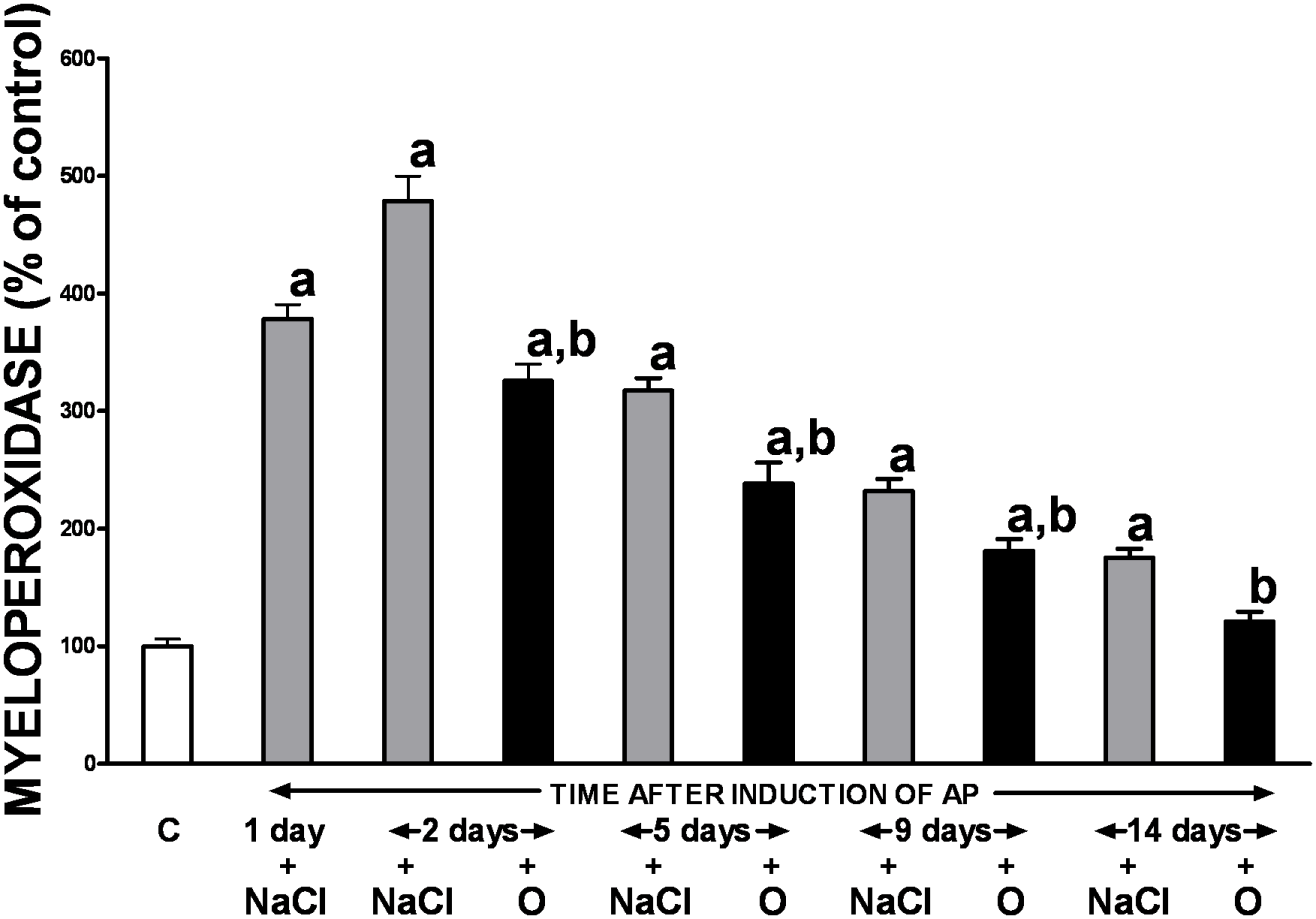
Influence of treatment with saline or obestatin on pancreatic activity of myeloperoxidase in the course of ischemia/reperfusion-induced acute pancreatitis. Key: C = control without induction of acute pancreatitis and treated with saline; O = obestatin given i.p. at the dose of 8 nmol/kg/dose twice a day, starting 24 h after induction of acute pancreatitis; NaCl = saline given i.p. twice a day, starting 24 h after induction of acute pancreatitis; AP = ischemia/reperfusion-induced acute pancreatitis. Values are expressed as mean ± SEM. ^a^P<0.05 compared to control; ^b^P<0.05 compared to NaCl-treated rats after induction of AP at the same time of observation.

Acute ischemia/reperfusion-induced pancreatitis significantly increased activity of myeloperoxidase in the pancreatic tissue, with the highest MPO level change (a 5-fold rise) seen on the day 2 (Fig 4). However, the observed alteration was not permanent and the gradual spontaneous normalization of pancreatic myeloperoxidase activity was seen over the time, beginning on the day 5. Administration of obestatin significantly accelerated reduction in MPO activity throughout the entire study.

### Pancreatic DNA synthesis

**Fig 5:**
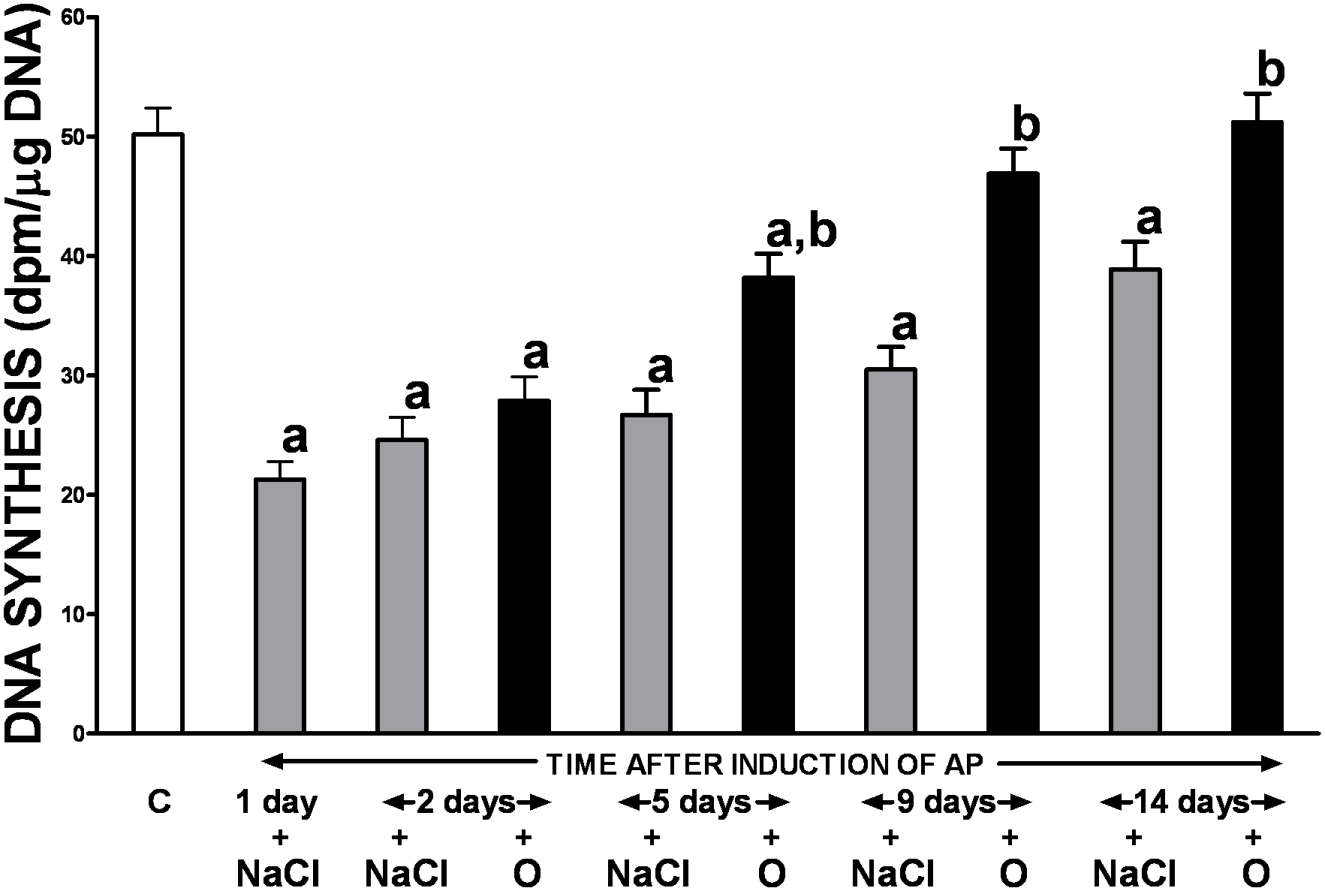
Influence of treatment with saline or obestatin on pancreatic DNA synthesis in the course of ischemia/reperfusion-induced acute pancreatitis. Key: C = control without induction of acute pancreatitis and treated with saline; O = obestatin given i.p. at the dose of 8 nmol/kg/dose twice a day, starting 24 h after induction of acute pancreatitis; NaCl = saline given i.p. twice a day, starting 24 h after induction of acute pancreatitis; AP = ischemia/reperfusion-induced acute pancreatitis. Values are expressed as mean ± SEM. ^a^P<0.05 compared to control; ^b^P<0.05 compared to NaCl-treated rats after induction of AP at the same time of observation.

Ischemia/reperfusion-induced AP resulted in the initial reduction of pancreatic cell vitality and proliferation, measured as a rate of pancreatic DNA synthesis, with subsequent gradual recovery during the study (Fig 5). Treatment with obestatin partly reversed the pancreatitis-evoked drop of pancreatic DNA synthesis and this effect was statistically significant between the 5^th^ and 14^th^ day of the study.

### Histological findings

**Fig 6:**
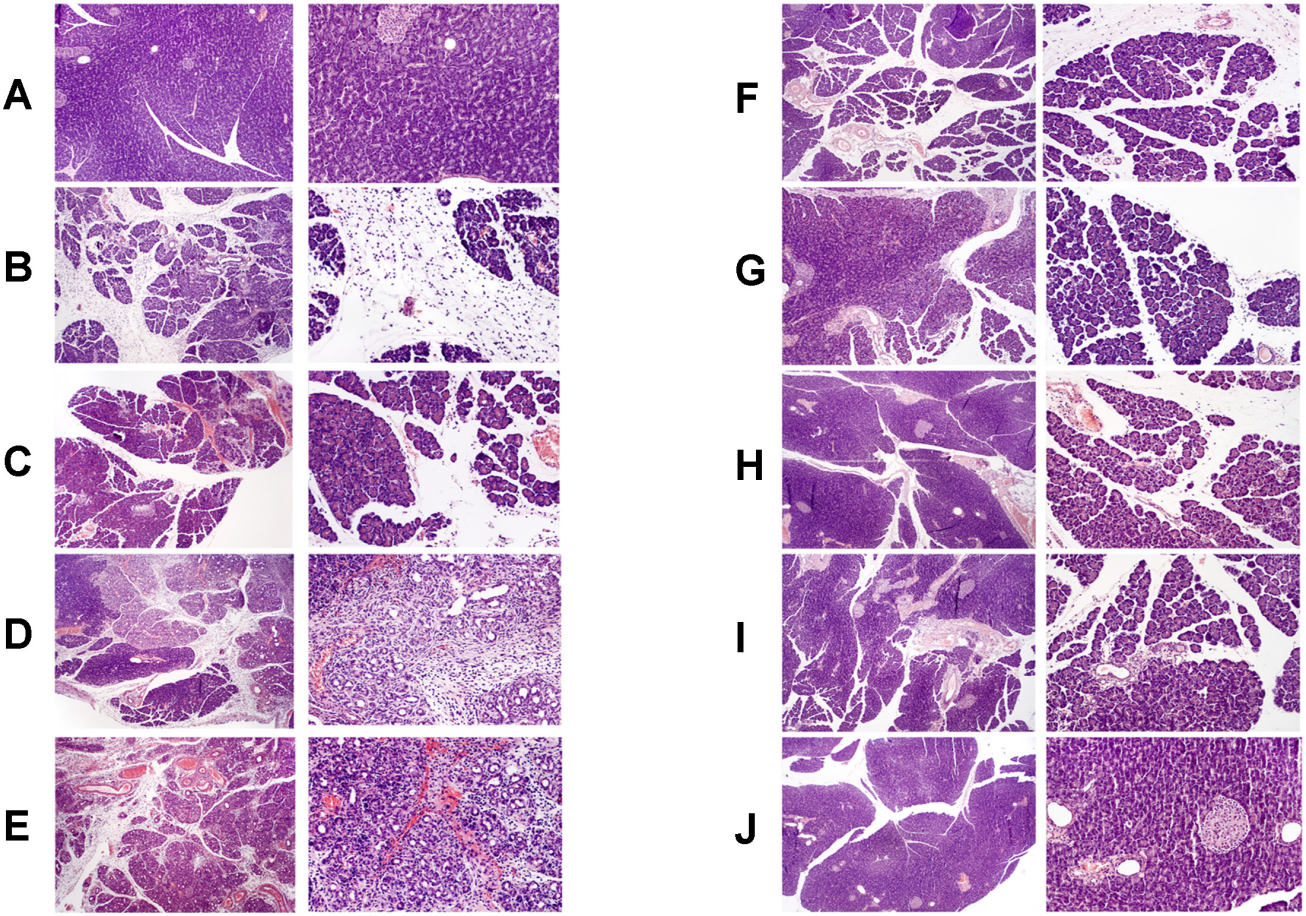
Representative morphological features of the pancreas. Hematoxilin-eosin counterstain. Histological images are presented in two different magnification for each group. In left column, original magnification 100×. In right column, original magnification 200×. (panel A) sham-operated control rats treated with saline; (panel B) rats with ischemia/reperfusion-induced pancreatitis after 1-day reperfusion; (panel C) rats with ischemia/reperfusion-induced pancreatitis after 2-days reperfusion and treated with saline; (panel D) rats with ischemia/reperfusion-induced pancreatitis after 2-days reperfusion and treated with obestatin; (panel E) rats with ischemia/reperfusion-induced pancreatitis after 5-days reperfusion and treated with saline; (panel F) rats with ischemia/reperfusion-induced pancreatitis after 5-days reperfusion and treated with obestatin; (panel G) rats with ischemia/reperfusion-induced pancreatitis after 9-days reperfusion and treated with saline; (panel H) rats with ischemia/reperfusion-induced pancreatitis after 9-days reperfusion and treated with obestatin; (panel I) rats with ischemia/reperfusion-induced pancreatitis after 14-days reperfusion and treated with saline; (panel J) rats with ischemia/reperfusion-induced pancreatitis after 14-days reperfusion and treated with obestatin. Saline or obestatin (8 nmol/kg/dose) were given i.p. twice a day, starting 24 h after induction of acute pancreatitis.

Histological scoring of pancreatic tissue damage in rats with or without ischemia/reperfusion-induced AP, and treated with either saline or obestatin is presented in Table 1. Pancreatic ischemia followed by reperfusion induced acute hemorrhagic pancreatitis in all tested animals. Twenty four hours after the beginning of reperfusion, at the light microscopic level, moderate inter- and intralobular edema was accompanied by moderate perivascular and scarce diffuse leukocyte infiltrations of the pancreatic tissue. Vacuolization was present in less than 25% of acinar cells. Necrosis involved less than 15–35% of pancreatic cells. In addition, 1–5 hemorrhagic foci per slide were observed in rats with AP (Table 1; Fig 6B). During the course of disease, the histological signs of tissue damage were spontaneously reduced and only interlobular edema, mild perivascular leukocyte infiltrations, and vacuolization present in less than 25% of acinar cells were observed at the 14^th^ day of pancreatic reperfusion (Table 1; Fig 6E, 6G and 6I). Treatment with obestatin accelerated pancreatic regeneration, and no microscopic signs of pancreatic injury, except the presence of mild perivascular leukocyte infiltrations, were observed in animals treated with the polypeptide at the last day of the experiment (Table 1, Fig 6J).

**Table 1:** Influence of treatment with saline or obestatin on histological signs of pancreatic damage in the course of ischemia/reperfusion-induced AP.

	EDEMA (0–3)	INFLAMMATORYINFILTRATION (0–3)	VACUOLIZATION (0–3)	NECROSIS (0–3)	HEMORRHAGES (0–3)
**CONTROL**	**0**	**0**	**0**	**0**	**0**
**AP 1 day**	**2**	**2**	**1**	**1–2**	**1–2**
**AP 2 days + Saline**	**2**	**2–3**	**1–2**	**1–2**	**2**
**IR 2 days + Obestatin**	**1–2**	**2**	**1**	**1**	**1–2**
**IR 5 days + Saline**	**1–2**	**2**	**1**	**1**	**0–1**
**IR 5 days + Obestatin**	**2–3**	**1–2**	**0**	**0**	**0**
**IR 9 days + Saline**	**1**	**2**	**0–1**	**0**	**0–1**
**IR 9 days + Obestatin**	**1**	**1**	**0**	**0**	**0**
**IR 14 days + Saline**	**1**	**1**	**0–1**	**0**	**0**
**IR 14 days + Obestatin**	**0**	**0–1**	**0**	**0**	**0**

Numbers represent the predominant histological grading in each experimental group. Key: Control = rats without induction of acute pancreatitis and treated i.p. with saline; Obestatin = rats treated with obestatin given i.p. at the dose of 8nmol/kg/dose twice a day, starting 24 h after induction of acute pancreatitis; NaCl = saline given i.p. twice a day, starting 24 h after induction of acute pancreatitis; AP = ischemia/reperfusion-induced acute pancreatitis.

### Food intake and secretory function

**Fig 7:**
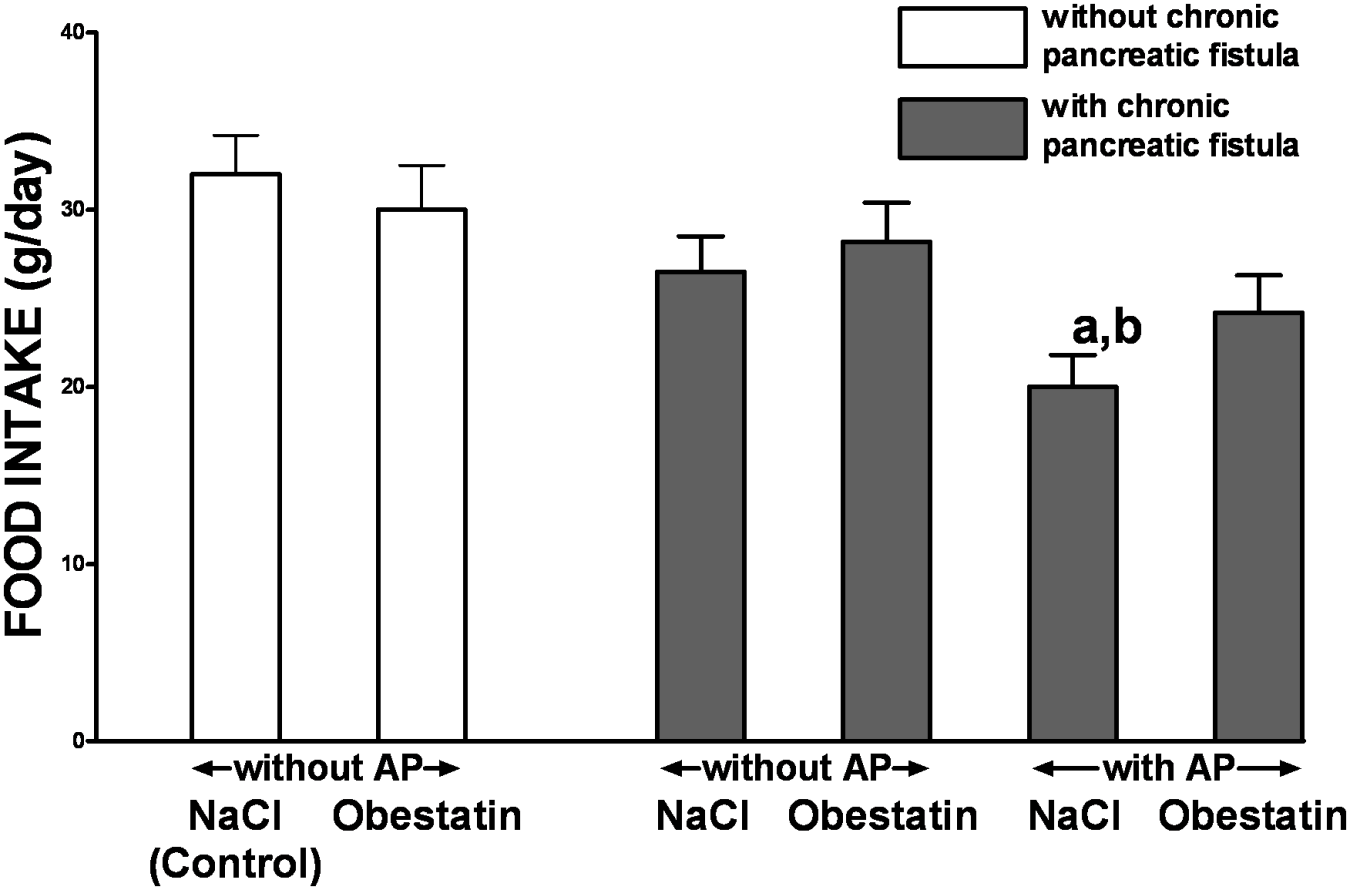
Influence of treatment with saline or obestatin and ischemia/reperfusion ischemia/reperfusion-induced acute pancreatitis on daily food intake in rats without or with implantation of chronic pancreatic fistula. Key: Control = rats without implantation of chronic pancreatic fistula and treated twice i.p. with saline during one-day observation; NaCl = rats treated with saline given twice i.p. during one-day observation; Obestatin = rats treated with obestatin given twice i.p. at the dose of 8 nmol/kg/dose during one-day observation; AP = ischemia/reperfusion-induced acute pancreatitis. Values are expressed as mean ± SEM. ^a^P<0.05 compared to control saline-treated rats; ^b^P<0.05 compared to obestatin-treated rats without implantation of chronic pancreatic fistula or induction of AP.

In control rats without chronic pancreatic fistula and treated with saline, daily food intake was 32.0 ± 2.2g per rat (Fig 7). In these rats, treatment with obestatin given at the dose 8nmol/kg/dose was without a significant effect on daily food intake. In rats without induction of AP, implantation of chronic pancreatic fistula tended to reduce daily food intake, but this effect was also statistically insignificant. On the other hand, daily food intake was significantly reduced in rats with AP induced after implantation of pancreatic fistula, when compared to control animals without pancreatic fistula and induction AP. In these rats, treatment with obestatin partly improved daily food intake and abolished a significant difference between this group of animals and control animals without pancreatic fistula and AP (Fig 7).

**Fig 8:**
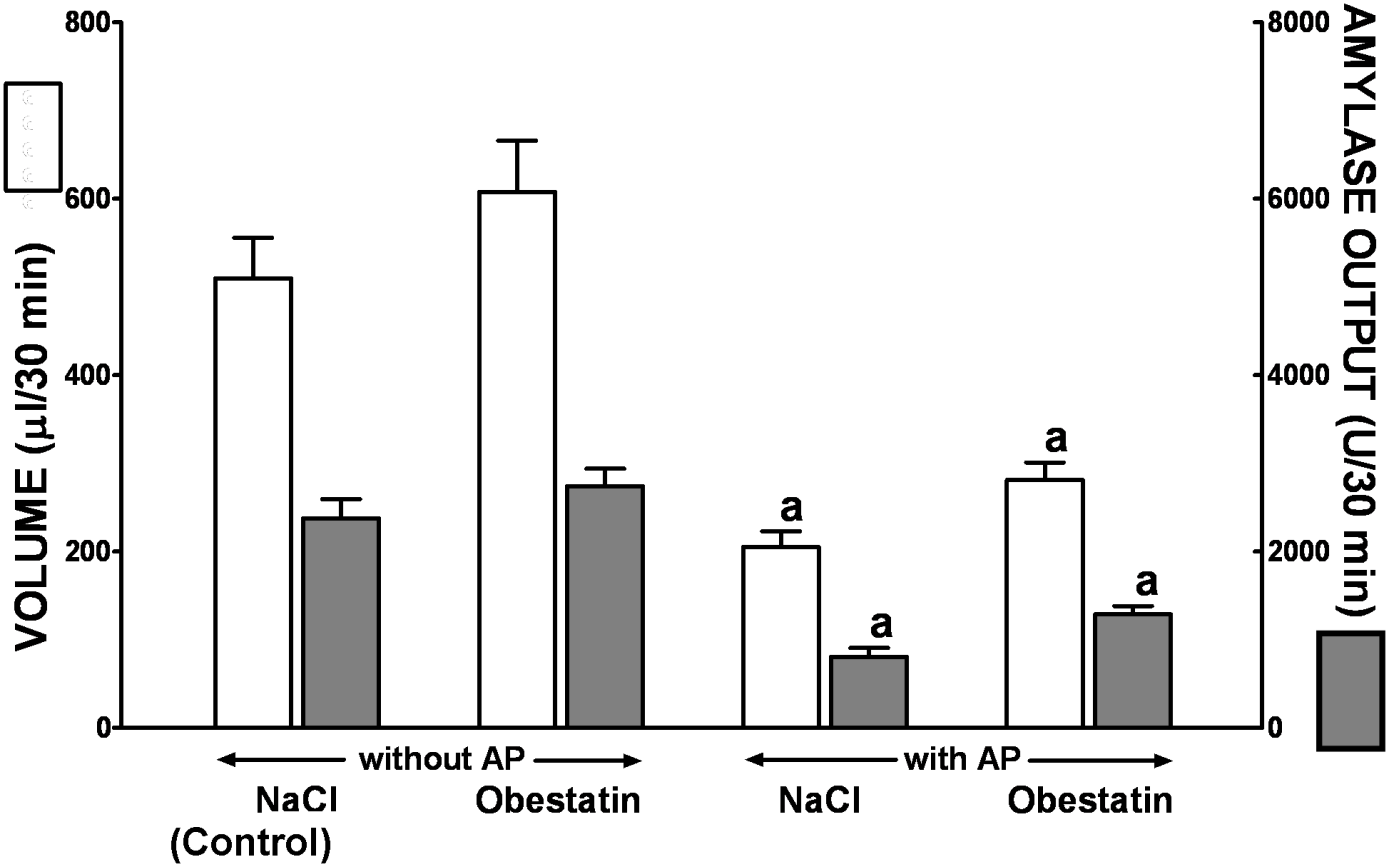
Volume of pancreatic secretion and amylase output under basal conditions in conscious rats without or with ischemia/reperfusion-induced acute pancreatitis and pretreated with saline or obestatin. Key: NaCl = rats pretreated with saline; Obestatin = rats pretreated with obestatin given twice a day i.p. at the dose of 8 nmol/kg/dose; AP = ischemia/reperfusion-induced acute pancreatitis. Values are expressed as mean ± SEM. ^a^P<0.05 compared to control saline-treated rats without induction of AP; ^b^P<0.05 compared to obestatin-treated rats without induction of AP.

In conscious control rats with chronic pancreatic fistula without induction of AP, a basal volume of pancreatic secretion and amylase output were 510 ± 46μl/30min and 2378 ± 215 U/30min, respectively (Fig 8). Induction of AP significantly reduced a volume of basal pancreatic secretion and amylase output by around 60 and 66%, respectively. Pretreatment with obestatin tended to increase, especially in rats with AP, a basal volume of pancreatic secretion and amylase output, but this effect was statistically insignificant (Fig 8).

**Fig 9:**
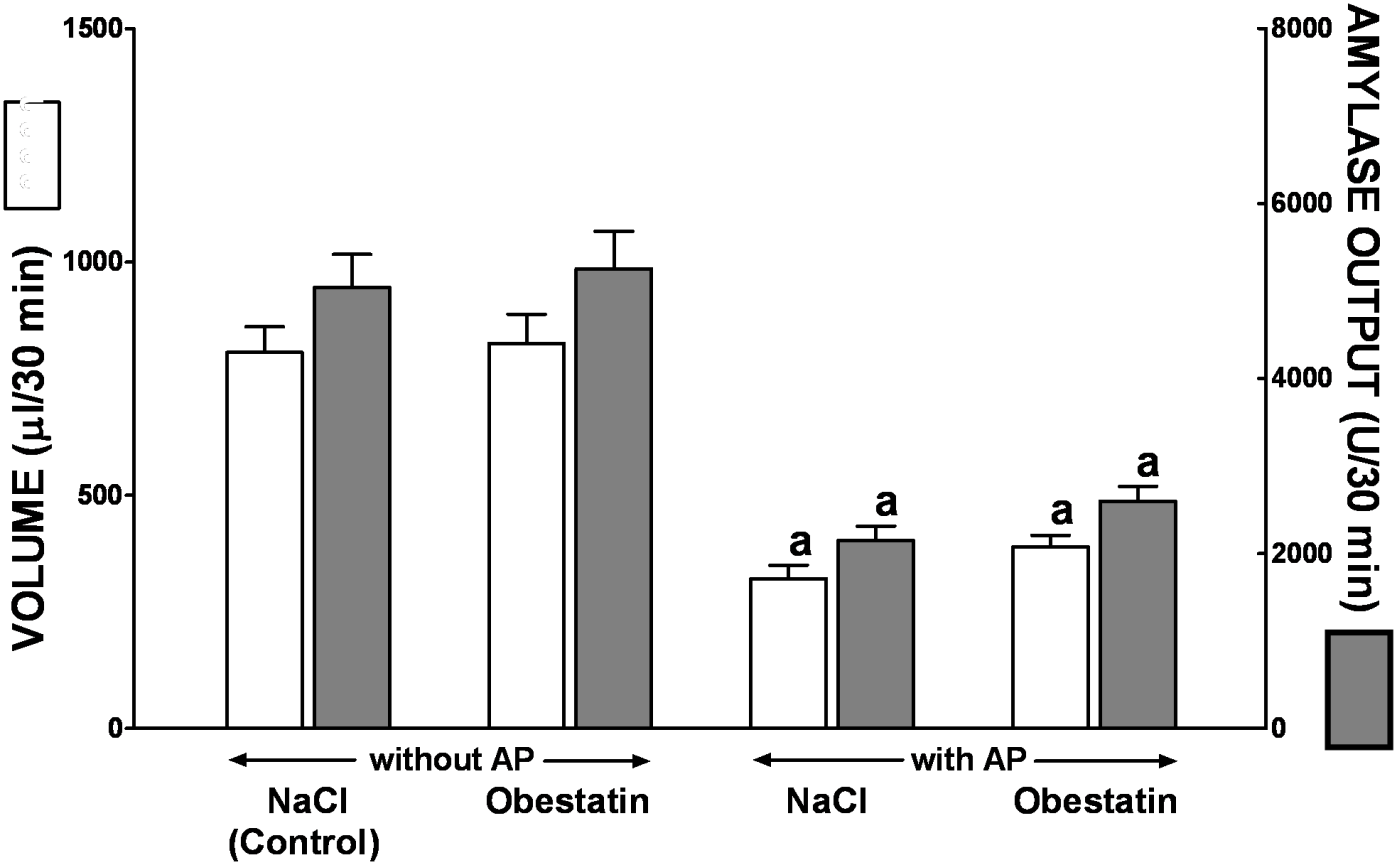
Volume of pancreatic secretion and amylase output following i.p. administration of cerulein given at the dose 1 μg/kg in conscious rats without or with ischemia/reperfusion-induced acute pancreatitis and pretreated with saline or obestatin. Key: NaCl = rats pretreated with saline; Obestatin = rats pretreated with obestatin given twice a day i.p. at the dose of 8 nmol/kg/dose; AP = ischemia/reperfusion-induced acute pancreatitis. Values are expressed as mean ± SEM. ^a^P<0.05 compared to control saline-treated rats without induction of AP; ^b^P<0.05 compared to obestatin-treated rats without induction of AP.

In control saline treated rats without induction of AP, following administration of cerulein given i.p. at the dose 1 μg.kg a volume of pancreatic juice and amylase output reached a value 806 ± 55μl/30min and 9453 ± 705U/30min, respectively (Fig 9). In saline-treated rats with AP, a volume of cerulein-stimulated pancreatic secretion and amylase output were reduced by around 60 and 57%, respectively, when compared to values observed in control rats without AP. Pretreatment with obestatin was without significant effect on the cerulein-stimulated pancreatic secretion in rat without or with AP (Fig 9).

## Discussion

Several previous studies have proved the protective and regenerative effects of the preproghrelin gene-derived peptides, ghrelin and obestatin in the gastrointestinal system, kidney and brain [10–12]. It has been also shown that ghrelin exerts a pronounced protective and therapeutic effect in the pancreas. Administration of ghrelin inhibits the development and accelerates the recovery in cerulein- and ischemia/reperfusion-induced AP [24–27]. Moreover there are studies indicating that pretreatment with obestatin inhibits the development of cerulein-induced [13] and ischemia/reperfusion-induced AP [28]. However, it must be pointed out that protective effect against the development of AP has only limited clinical value. Preventive effect requires pretreatment with medicine before exposure to factors leading to the development of the disease. Typically patient with AP are seen in the hospital several hours or even days after the onset of illness. For this reason, in our current study, we investigated the impact of obestatin treatment on the course of ischemia/reperfusion-induced AP. To our knowledge, the present study is the first report that the administration of obestatin exhibits a therapeutic effect in the course of necrotizing AP. This effect has been found as an improvement of functional, biochemical and histological parameters of pancreatic condition.

Pancreatitis is caused by inflammatory injury to the exocrine pancreas, from which recovers via regeneration of digestive enzyme-producing acinar cells. This regenerative process involves transient phases of inflammation, metaplasia, and redifferentiation, driven by cell-cell interactions between acinar cells, leukocytes, and resident fibroblasts [29].

Our present studies have shown that treatment with obestatin expedites spontaneous normalization of pancreatic structure in the course of ischemia/reperfusion-induced AP leading to earlier reduction and/or elimination of pancreatic edema, leukocyte infiltrations of the pancreatic tissue, vacuolization of acinar cells, pancreatic necrosis and number of hemorrhagic foci. Administration of obestatin has accelerated tissue regeneration, and no microscopic signs of pancreatic injury except the presence of mild perivascular leukocyte infiltrations were observed in animals treated with the polypeptide on the last day of the experiment.

The obestatin-related reduction in the inflammatory leukocyte infiltration of pancreatic tissue was orchestrated with a precipitated decrease in the gland myeloperoxidase (MPO) activity in animals with AP treated with the polypeptide compared to those receiving saline only. MPO is considered to be a marker of local neutrophil activity causing tissue damage in various inflammatory diseases including AP [30, 31]. In addition, in patients with AP, it has been shown that MPO contributes to the production of reactive oxygen metabolites and its level depends on the severity of AP, as well as on cytokine blood level [31]. Results of the present study remain in concordance with the previous observation that treatment with obestatin leads to the suppression of neutrophil accumulation and reduction in ischemia/reperfusion-induced renal injury [11].

Interleukin-1β (IL-1β) is a well-known mediator of acute inflammation which plays a crucial role in the release of other members of pro-inflammatory cytokine cascade including tumor necrosis factor alpha, platelet-activating factor, prostaglandins and pro-inflammatory interleukins, and consequently stimulates the development of AP [32, 33]. Moreover, it has been demonstrated that early and sustained activation of inflammatory cells with successive release of IL-1β and other cytokines is responsible for the intense local and systemic inflammatory response in AP, as well as, the development of chronic inflammation and fibrosis of the gland [34]. Contrarily, inhibition of the cytokine cascade at the level of the IL-1 receptor, before or soon after induction of pancreatitis, markedly attenuates the rise in these cytokines and is associated with decreased severity of pancreatitis and reduced pancreatic damage [32, 35]. In the present study, similar to other reports [13, 25, 26], we have detected the increase in serum levels of IL-1β in rats after induction of acute necrotizing pancreatitis followed by spontaneous gradual normalization of the cytokine serum concentration over the time. Importantly, treatment with obestatin precipitated that process limiting, therefore, the severity of the disease. This vital finding indicates one of mechanisms involved in the therapeutic effect of obestatin in ischemia/reperfusion-induced AP, and it is in line with the previous report that protective effect of pretreatment with obestatin in cerulein-induced AP is associated with a reduction in serum level of IL-1β [13].

Further supporting evidence of the therapeutic properties of obestatin in the course of ischemia/reperfusion-induced AP is related to the attenuation of serum lipase activity. This enzyme is released by acinar cells to the interstitial tissue during AP, and its concentration in serum serves as an index of AP severity with high sensitivity and specificity [34, 36]. In our experiment 24 h after the start of pancreatic reperfusion, rats with ischemia/reperfusion-induced AP demonstrated a 10-fold increase in serum lipase activity compared to the controls. The observed detrimental effect was temporary and the gradual spontaneous decrease in serum enzyme activity was observed throughout the study, starting on day 2 of the experiment. Treatment with obestatin accelerated normalization of serum lipase activity.

Healing of injured organs requires cell proliferation. The rate of pancreatic DNA synthesis serves as a measure of cell vitality and proliferation. Several previous studies have demonstrated that AP, irrespective of etiology, may lead to initial inhibition of pancreatic DNA synthesis followed by a subsequent increase in this parameter [25, 37–39]. Our results confirmed that pattern in the ischemia-reperfusion AP model in which the severity of pancreatic injury was very pronounced and pancreatic cell proliferation was initially reduced by approximately 80% and was followed by a gradual spontaneous improvement in DNA synthesis. Importantly, the gland healing was expedited by administration of obestatin. Therefore, our findings further corroborate the therapeutic effect exerted by obestatin in AP.

Circulation and perfusion of tissues are essential physiological processes necessary to provide and sustain oxygenation and nutrition at a cellular level. It has been shown that the initial damage of postischemic pancreas is mainly characterized by microcirculatory dysfunction [40, 41]. Moreover, the propagation of AP is associated with microvascular impairment of the gland with subsequent formation of thrombi in capillaries, activation of leukocytes and release of proteolytic enzymes and-pro-inflammatory cytokines [42, 43]. Furthermore, tissue damage due to microcirculation failure in the course of AP often affects distant organs including the kidney, colon, liver and lungs [44]. In contrast, the improvement in pancreatic blood flow [45, 46], as well as the anticoagulation treatment [47] inhibit the development of AP and precipitate tissue regeneration in the course of the disease. Consistent with the aforementioned reports, our study confirmed a deleterious impact of acute pancreatitis on the gland perfusion. However, this effect was not permanent and pancreatic blood flow spontaneously improved reaching nearly 80% of baseline value on day 14. Importantly, obestatin treatment resulted in significant expedition of tissue blood flow recovery observed since the 5^th^ day of AP, with almost full recuperation achieved 9 day later. This observation remains in line with the previous study demonstrating the improvement in pancreatic blood flow in rats with cerulein-induced acute pancreatitis pretreated with obestatin [13], and corroborates further the universal therapeutic impact of obestatin in AP. Although the exact mechanisms through which obestatin can protect and restore tissue blood flow in acutely inflamed pancreas remains unknown it has recently been shown that obestatin along with other preproghrelin gene-derived peptides demonstrates antiapoptotic actions in human pancreatic islet microendothelial cells exposed to chronic hyperglycemia, and the effects and signaling mechanisms induced by the peptides are comparable and also similar to those of the GLP-1R agonist exendin-4 [48]. On the other hand, previous studies have shown that genetic and pharmacological interference with GLP-1R does not affect the severity of pancreatitis in cerulein model of this disease. This last observation suggests that therapeutic effect of obestatin in ischemia/reperfusion-induced AP is probably independent to GLP-1R signaling [49].

In 2005, Zhang et al. [50] isolated a new 23-amino-acid peptide derived from prepro-ghrelin and named it obestatin a contraction of obese, from the Latin "obedere," meaning to devour, and "statin," denoting suppression. They have reported that contrary to the appetite-stimulating effects of ghrelin, obestatin reduces food intake in rats. However, the effect of obestatin on food intake is controversial. Some studies have conformed anorexigenic effect of obestatin [7, 51], whereas other studies have stated that obestatin does not show any effect on food intake in mice and rats [7, 51]. According to those last reports, we have found in our current study that treatment with obestatin given at the dose 8nmol/kg/dose was without a significant effect on daily food intake in rats without induction of AP. On the other hand, induction of AP significantly reduced daily food intake and administration of obestatin in this group of animals partly improved food intake and abolished a significant difference between animals with AP and control animals without pancreatic fistula and induction of AP. This observation may suggest that partial reversion of the pancreatitis-evoked decrease in food intake after administration of obestatin is not a result of direct action of obestatin on feeding behavior, but the effect of improvement in pancreatic condition.

In our present study we have found that induction of AP significantly reduces pancreatic a volume of pancreatic exocrine secretion amylase output in basal condition and after stimulation by cerulein. This observation is in agreement with numerous previous reports showing inhibition of pancreatic secretion in acute pancreatitis [52, 53]. Our present study has also bring an observation that obestatin administered at the dose of 8nmol/kg/dose does not significant affect pancreatic exocrine secretion on rats with or without induction of acute pancreatitis. This observation is in partial disagreement with previous studies performed by Kapica et al. [54]. They have shown that intravenous or intraduodenal administration of obestatin stimulates pancreatic protein and trypsin output in anesthetized rats. However, it must be pointed out that Kapica et al. used obestatin at the extremal high doses, such as 30, 100 and 300 nmol/kg/dose [54]. On the other hand, a lack of pancreatic secretion reduction after administration of obestatin in our present study indicates that therapeutic effect of obestatin in the course of ischemia/reperfusion-induced AP is not related to inhibition of pancreatic secretion.

In conclusion, obestatin treatment has attenuated the severity of AP, and facilitated both functional and structural recovery in a rat model of ischemia/reperfusion-induced AP. The mechanisms involved are likely multifactorial and the therapeutic effects of obestatin seem to be related, at least in part, to the inhibition of the inflammatory process in the pancreas, and the improvement of pancreatic DNA synthesis, and pancreatic blood flow. This observation suggests that treatment with obestatin may be useful in the therapy of AP.
